# Unravelling the genome of long chain *N*-acylhomoserine lactone-producing *Acinetobacter* sp. strain GG2 and identification of its quorum sensing synthase gene

**DOI:** 10.3389/fmicb.2015.00240

**Published:** 2015-04-14

**Authors:** Kah Yan How, Kar-Wai Hong, Choon-Kook Sam, Chong-Lek Koh, Wai-Fong Yin, Kok-Gan Chan

**Affiliations:** ^1^Division of Genetics and Molecular Biology, Institute of Biological Sciences, Faculty of Science, University of MalayaKuala Lumpur, Malaysia; ^2^Natural Sciences and Science Education Academic Group, National Institute of Education, Nanyang Technological UniversitySingapore, Singapore

**Keywords:** *Acinetobacter*, *N*-acylhomoserine lactone, AHL synthase, liquid chromatography–mass spectrometry (LC–MS), quorum sensing, protein expression

## Abstract

Myriad proteobacteria use *N*-acyl homoserine lactone (AHL) molecules as quorum sensing (QS) signals to regulate different physiological functions, including virulence, antibiotic production, and biofilm formation. Many of these proteobacteria possess LuxI/LuxR system as the QS mechanism. Recently, we reported the 3.89 Mb genome of *Acinetobacter* sp. strain GG2. In this work, the genome of this long chain AHL-producing bacterium was unravelled which led to the molecular characterization of *luxI* homologue, designated as *aciI*. This 552 bp gene was cloned and overexpressed in *Escherichia coli* BL21(DE3). The purified protein was ∼20.5 kDa and is highly similar to several autoinducer proteins of LuxI family among *Acinetobacter* species. To verify the AHL synthesis activity of this protein, high-resolution liquid chromatography–mass spectrometry analysis revealed the production of 3-oxo-dodecanoyl-homoserine lactone and 3-hydroxy-dodecanoyl-homoserine lactone from induced *E. coli* harboring the recombinant AciI. Our data show for the first time, the cloning and characterization of the *luxI* homologue from *Acinetobacter* sp. strain GG2, and confirmation of its AHLs production. These data are of great significance as the annotated genome of strain GG2 has provided a valuable insight in the study of autoinducer molecules and its roles in QS mechanism of the bacterium.

## Introduction

It has long been appreciated that bacteria communicate with each other using chemical signal molecules. These molecules are critical in coordinating gene expression and synchronizing the activities of the entire community. The term “quorum sensing” (QS) refers to regulation of gene expression in response to fluctuations in cell-population density. QS bacteria constitutively produce, release, detect, and respond to chemical signaling molecules called autoinducers which generally accumulate as the cells grow in number (Fuqua et al., 2001; Miller and Bassler, 2001; Schauder and Bassler, 2001). Depending upon the bacterial species, various physiological processes are mediated by these cell–cell communication systems, including antibiotic production, virulence, symbiosis, conjugation, and biofilm formation (Schauder and Bassler, 2001).

In the past decades, one of the most well-studied QS signaling molecules is *N*-acyl homoserine lactone (AHL) which is mainly found in Gram-negative bacteria (Williams et al., 2007). AHL molecules are highly conserved among bacteria. Each AHL molecule consists of a homoserine lactone ring unsubstituted in the β- and γ-positions but *N*-acylated at the α-position with a fatty acyl group. The structure of AHL shows variation in acyl chain length (varies from C4 to C18), the degree of saturation, and the oxidation states (presence of a hydroxy-, oxo-, or no substituent at the C3 position; Chhabra et al., 2005). Hence, it is the fatty acyl group that confers QS signal specificity among the bacteria (Cooley et al., 2008). AHL molecules are synthesized by LuxI homologue synthase. When AHL concentration has reached its threshold level, the AHLs will bind to their cognate receptor (LuxR homologue), thereby stimulating the expression of numerous downstream target gene. Each LuxR-type protein is highly specific for its respective AHL. There are multiple QS circuits that control a myriad of specific genes that express many bacterial phenotypes and potential virulence determinants (Parsek and Greenberg, 2000).

Dong et al. (2002) reported that the biochemical mechanism of action of the LuxI/LuxR pairs is conserved in many bacterial species. In most cases, further regulatory complexity has been added to the basic biochemical mechanism. This allows a multitude of behaviors to be functioned and controlled by a common mechanism. This can be seen in opportunistic human pathogen *Pseudomonas aeruginosa* in which two LuxI/R pairs were found (called LasI/LasR and RhlI/RhlR) that work simultaneously to control the expression of genes involved in biofilm formation and virulence (Passador et al., 1993; Brint and Ohman, 1995; Glessner et al., 1999). Besides its role in many physiological processes, the bacterial QS is also essential in developing pathogenic relationships with eukaryotic hosts. Hence, the AHL signaling system has been regarded as a promising target for developing novel approaches to interfere with microbial QS by regulating the virulence of the entire population. This indirectly creates a less selection pressure for the evolution of antibiotic-resistance in the bacteria (Hentzer and Givskov, 2003).

The genus *Acinetobacter* comprises aerobic, Gram-negative, non-fermentative bacteria that are isolated from diverse environments (Bhargava et al., 2010). As of now, at least 14 complete genomes of *Acinetobacter* species are available in the database, and only three environmental isolates have been sequenced (Kim and Park, 2013). A flurry of research over the past decade has focused mainly on *A. baumannii* strains because of their clinical importance as the primary pathogenic bacteria in nosocomial infections. Hence, the role of QS system in soil-borne or plant-associated *Acinetobacter* sp., which have been only rarely explored, are a valuable line of study.

Our group have been exploring rhizosphere environment for bacterial communities in the Malaysian rainforest. Recently, we isolated *Acinetobacter* sp. strain GG2 from rhizosphere of ginger (*Zingiber officinale*; Chan et al., 2009, 2011) and its genome was fully sequenced by Illumina platform (Hong et al., 2012) and deposited in GenBank. This soil isolate was found to secrete only long chain AHLs, particularly 12-carbon in acyl chain length with different variant. In the present study, we aimed to analyze the genome assembly for gene predictions and annotations, as well as comparative genome analysis with other closest sequenced *Acinetobacter* spp. The annotated genome led us to the investigation of the putative homologue of AHL synthase, designated as *aciI*. The *aciI* gene was cloned and overexpressed in *Escherichia coli* and the purified protein was characterized. Mass spectrometry confirmed the production of AHLs was directed by the recombinant AciI protein.

With the availability of the whole-genome and AciI of isolate GG2, these genome data will path the way for functional study of QS in GG2 in the future. As such, the verification of the synthase activity provides a platform to study the regulatory role of the AHLs on virulence and unknown genetic traits of the soil-dwelling bacterium. In addition, *Acinetobacter* species isolated from diverse environments may have profound biotechnological potential such as degradation of a variety of pollutants as they appear to be metabolically versatile (Jung et al., 2010). As the frequency of multidrug-resistance among *Acinetobacter* strains is increasing (Bhargava et al., 2010), QS as the global regulator is gaining more importance as the target for antimicrobial strategies to attenuate bacterial virulence. It is reported that within the genus, some of the *Acinetobacter* environmental species are genetically closely related to the clinical sources (Gonzalez et al., 2009). Hence, this facilitates the study of QS mechanism in environmental strains of *Acinetobacter* sp. which could also be applied to the pathogenic isolates.

## Materials and Methods

### Bacterial Strains and Culturing Conditions

*Acinetobacte*r sp. strain GG2 was isolated from the rhizosphere of ginger (*Z. officinale*) from the Malaysian tropical soil. The bacteria were grown aerobically in Luria-Bertani (LB) medium or LB agar (Merck, Germany) at 25°C in shaking incubator (220 rpm). *E. coli* DH5α (Invitrogen, USA) and BL21 (DE3) pLysS (Novagen, Germany) were used to propagate the recombinant plasmids and to overexpress the fusion protein, respectively. For *E. coli* strains, the bacteria were grown in LB medium at 37°C with shaking. The transformed cells were grown in LB supplemented with 100 μg/ml ampicillin (Sigma, St. Louis, MO, USA), 30 μg/ml kanamycin (Sigma, St. Louis, MO, USA) or 34 μg/ml chloramphenicol (Sigma, St. Louis, MO, USA), if necessary. All the bacterial isolates were stored frozen at –70°C.

### Isolation of Genomic DNA

Briefly, a single colony of strain GG2 was inoculated into 10 ml of LB medium and it was grown overnight. From this overnight-grown culture, 1 ml was taken and harvested. The pellet was lysed with DNAzol reagent (Invitrogen, USA) followed by addition of Proteinase K (NEB, USA) and incubation for 3 h. To precipitate the DNA, absolute ethanol was then added to the lysate. The resulting DNA pellet was washed twice with 75% (v/v) ethanol and air-dried. Following this, the DNA was dissolved in TE buffer (pH 8.0) and stored at 4°C. Plasmid DNA for use in subcloning was isolated using QIAprep Spin Miniprep Kit (Qiagen, Germany) according to manufacturer’s instructions. The purity of the DNA was estimated using spectrophotometry by calculating the A260/280 ratio, and the yield was determined using Qubit 2.0 fiuorometer (Life Technologies, Carlsbad, CA, USA).

### Construction of Recombinant AciI Expression Plasmids

The total genomic DNA of strain GG2 was used to amplify the *aciI* gene by PCR. Primers with the following sequences were used: G2-F (5′-CCATGGACATGAATATTATTGCTGGA-3′) and G2-R (5′-CTCGAGCACTTCAATCAAGCATG-3′). Two non-specific bases and an NcoI restriction site (underlined) were added to the forward primer. As for reverse primer, an XhoI restriction site (underlined) was added. In addition, the stop codon was removed to allow expression of C-terminal His-tag. The PCR cycles used consisted of an initial denaturation at 95°C for 5 min, followed by 30 cycles of 95°C for 30 s, annealing at 60°C for 30 s and extension at 72°C for 1 min, and a final extension at 72°C for 5 min. Sterile deionized water was used as the negative control. Agarose gel electrophoresis was employed to verify the size of the amplicon. Then, the PCR product was gel purified using QIAquick Gel Extraction kit (Qiagen, Germany) and ligated to pDrive cloning vector (Qiagen, Germany), according to the manufacturer’s instructions. The resulting recombinant plasmid (designated pDrive-aciI) was transformed into *E. coli* DH5α (Sambrook and Russel, 2001). The *aciI* gene was excised from this plasmid by digestion with NcoI and XhoI (Promega, USA) followed by gel purification, and ligated into pET28a (Novagen, Germany) digested with the same enzymes. This produced the recombinant plasmid designated pET28a-aciI. The sequence of *aciI* cloned into pDrive and pET28a plasmids was verified by automated Sanger DNA sequencing.

### Nucleotide Sequence and Bioinformatics Analysis

The nucleotide sequences of *aciI* were compared with GenBank databases using BLASTX program available from NCBI website^1^. Ten LuxI homologues with identities of ∼95% to AciI in terms of amino acid sequence were selected from the protein database. Redundant sequences or bacteria strain with ambiguities were omitted. All parameters were set at default and BLOSUM62 was employed in the scoring matrix. Searches for ORF and prediction of nucleotide translational products were performed using the ORF Finder tool^2^ while the fundamental properties of the proteins were predicted using by ExPASy^3^. Multiple sequence alignments of the amino acid sequences were carried out using Clustal OMEGA tool^4^ with its default parameter settings. A phylogenetic tree of the *aciI* gene was then constructed using molecular evolutionary genetics analysis (MEGA) version 5 (Tamura et al., 2011) using Neighbor-Joining strategy as described previously (Chan et al., 2010). Glucose dehydrogenase enzyme (GI: 729809030) from *E. coli* was chosen as the outgroup for comparison. Bootstrap analyses for 1,000 re-samplings were applied to provide confidence estimates for tree topologies while other parameters were maintained as default.

### Gene Annotation and Comparative Genome Analysis

From the draft genome of strain GG2, gene prediction was performed using Prodigal (v2.60; Hyatt et al., 2010) and gene annotation was performed using Blast2GO (Conesa et al., 2005). Further annotation was obtained using the SEED-based automated annotation system provided by the rapid annotations using subsystems technology (RAST) server (version 4.0; Aziz et al., 2008). Comparative analysis of the GG2 genome with the closest related species, *A. baumannii* AB0057 genome was performed using Mauve software (Darling et al., 2004), an online Java-based tool for ordering contigs and inspecting assembly statistic. BRIG (BLAST Ring Image Generator; Alikhan et al., 2011) software was used for the circular representation of multiple genome comparison among strain GG2 and other closely related species. The genome of strain GG2 was used as the reference genome and was compared with genome of *A. baumannii* AB0057 (CP001182), *A. baumannii* AYE (NC_010410), *A. baumannii* ACICU (CP000863), and *A. baumannii* ATCC 19606 (NZ_ACQB00000000.1) which were obtained from NCBI database.

### Heterologous Expression of AciI Protein in *E. coli*

His-tagged fusion expression plasmid, pET28a-aciI was transformed into *E. coli* BL21 (DE3)pLysS cells (Sambrook and Russel, 2001) and the transformants were selected on LB medium supplemented with kanamycin and chloramphenicol. After the desired clone was verified, 1 ml of the overnight culture was inoculated into 50 ml of LB medium containing both antibiotics and cells were grown at to 37°C until it reached OD_600_ of 0.4–0.5. Following this, isopropyl-D-thiogalactopyranoside (IPTG, Sigma, St. Louis, MO, USA) at final concentration of 1.0 mM was added to induce the expression of the *aciI* gene in *E. coli*. The induced culture was allowed to grow for 8 h at 25°C with shaking. *E. coli* harboring pET28a alone was used as the negative control. The cells were then harvested by centrifugation at 10,000 × *g* and lysed by BugBuster^TM^ Protein Extraction Reagent supplemented with protease inhibitors (Novagen, Germany). The recombinant proteins were purified from cell lysate using HisTrap^TM^ HP Column (GE Healthcare Lifesciences, Sweden) according to manufacturer’s instructions. This affinity column is prepacked with nickel sepharose to facilitate purification of His-tag proteins.

### Polyacrylamide Gel Electrophoresis (SDS-PAGE) Analysis

After IPTG induction, the bacteria cultures were harvested and the pellets were suspended in 2× Laemmli sample buffer (Bio-Rad, USA). The samples were boiled for 5 min before loaded into 12.5% (w/v) polyacrylamide gel electrophoresis system (PAGE, Bio-Rad, USA) in the presence of sodium dodecyl sulfate (SDS) according to the methods established by Laemmli (1970). To identify the protein bands, the gels were stained with Coomassie brilliant blue R-250 (CBB; Bio-Rad, USA).

### Extraction of AHL

The extraction of AHL from bacterial culture supernatants was performed using methods established previously with minor modifications (Chong et al., 2012). The induced *E. coli* BL21 cells harboring pET28a-aciI was grown in LB medium buffered to pH 6.5 with 50 mM of 3-[*N*-morpholino] propanesulfonic acid (MOPS) to prevent degradation of AHL (Yates et al., 2002). The spent culture supernatant was extracted thrice with equal volume of acidified ethyl acetate (0.1% v/v glacial acetic acid in ethyl acetate, Merck, Germany) and the organic solvent was evaporated to dryness in fume hood. The dried extracts were then resuspended in 1 mL of acidified ethyl acetate and allowed to dry again in fume hood. Finally, 1 ml of acetonitrile (HPLC grade, Merck, Germany) was added to dissolve the extracted AHL. The mixture was then centrifuged at maximum speed for 5 min to remove insoluble residues. From the top layer of the extracts, 100 μL of aliquots were withdrawn and placed in sample vials for analysis by liquid chromatography–mass spectrometry (LC–MS/MS).

### Identification of AHL by Mass Spectrometry (MS)

The profile of the extracted AHL was identified by high resolution tandem triple quadrupole mass spectrometry (LC-MS/MS). System according to previously reported method (Wong et al., 2012). Agilent 1290 Infinity system (Agilent Technologies Inc., Santa Clara, CA, USA) was employed as the LC delivery system coupled with Agilent ZORBAX Rapid Resolution HT column (2.1 mm × 50 mm, 1.8 μm particle size). Both mobile phases A and B were 0.1% v/v formic acid in water and 0.1% v/v formic acid in acetonitrile, respectively. The parameters of the gradient profiles were indicated as followed (time: mobile phase A: mobile phase B): 0 min: 60:40, 5 min: 20:80, 7 and 10 min: 5:95, and 11 and 13 min: 60:40. The injection volume was 2 μL and the analysis was performed using a flow rate of 0.3 mL/min at 37°C. The Agilent 6490 Triple-Quad LC-MS/MS system was used to perform the high-resolution electrospray ionization mass spectrometry (ESI-MS) in positive mode. The probe capillary voltage was set at 3 kV, sheath gas at 11 mL/h, nebulizer pressure at 20 psi and desolvation temperature at 250°C. Nitrogen gas was used as the collision gas in the collisionally induced dissociation mode for the MS/MS analysis and the collision energy was set at 10 eV. The Agilent MassHunter software was used to analyze the MS data (Wong et al., 2012; Yin et al., 2012). In addition, known amounts of synthetic AHLs (Sigma, St. Louis, MO, USA) were loaded as standards. The putative AHLs secreted by strain GG2 were detected with a breakdown product ion of *m/z* of 102. Acetonitrile and AHL extracted from culture supernatant of *E. coli* harboring pET28a alone were used as the blank and negative control, respectively.

## Results

In previous work by Hong et al. (2012), the Illumina HiSeq 2000 platform (Illumina Inc., San Diego, CA, USA) was employed to perform the whole-genome sequencing of strain GG2. The genome sequence has been deposited at DDBJ/EMBL/GenBank under the accession no. ALOW00000000. With approximately 56 fold coverage, the genome assembly generated 57 contigs with a total of 3,890,805 bp. The genome has G+C content of 38.4%. From Prodigal analysis, a total of 3,572 coding DNA sequences (CDS) were predicted and the genome coding density is 88%.

Annotations by RAST revealed a total of 419 subsystems with 48% subsystem coverage. A subsystem represents a collection of functional roles that make up a metabolic pathway, a multi-subunit complex (e.g., the ribosome) or a specific class of proteins (e.g., signal transduction). Meanwhile, subsystem coverage shows the percentage of the FIGfams (a set of proteins that are “globally similar” and in which all members share a common function) that is covered by subsystems (Aziz et al., 2008). Among the features of the subsystems, at least two third encode the basic core functions and metabolic pathways of the organism. The most abundant of the subsystems are related to amino acids and derivatives (*n* = 441, 16.8 of total subsystem features), followed by carbohydrates (*n* = 272, 10.4%), cofactors, vitamins, prosthetic groups, pigments (*n* = 226, 8.6%), protein metabolism (*n* = 209, 8.0%), RNA metabolism (162, 6.2%), and fatty acids, lipids and isoprenoids (154, 5.9%). From the analysis, other closely related *A. baumannii* species were also found to have high abundance of CDS coding for carbohydrate and amino acids and derivatives (Table S1). In addition, RAST annotation shows that *A. baumannii* AB0057 (score 537), *A. baumannii* AYE (score 532), *A. baumannii* ACICU (score 530), and *A. baumannii* ATCC 19606 (score 516) are the closest neighbors of the strain GG2.

**FIGURE 1:**
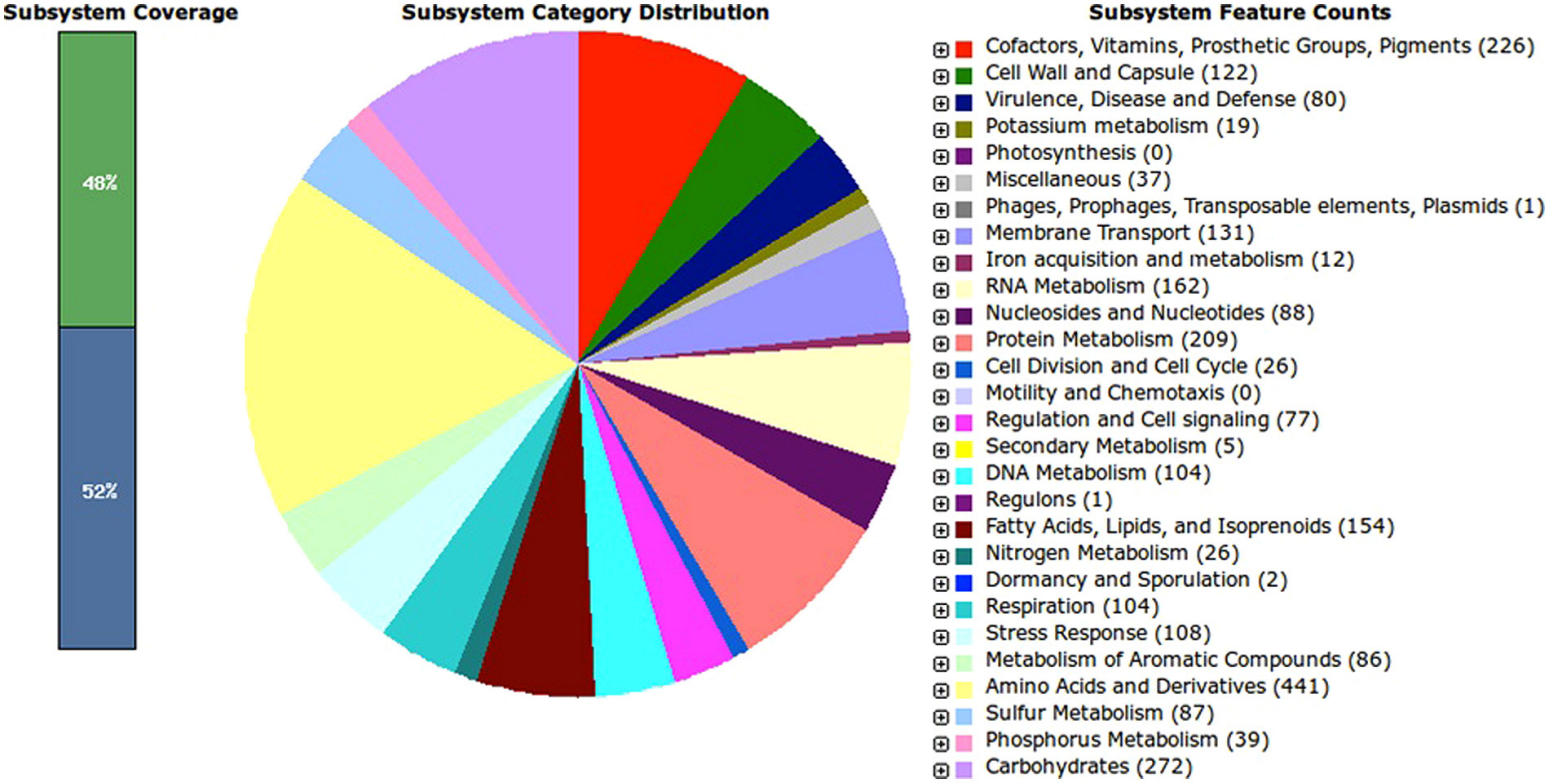
**Subsystem distribution of *Acinetobacter* sp. strain GG2 based on RAST annotation server.** Out of 3574 CDS predicted by RAST server, the subsystem coverage is 48% which contributes to a total of 419 subsystems. The green bar of the subsystem coverage indicates the percentage of the proteins included in the subsystems while the blue bar refers to the percentage of the proteins that are not included in the subsystems.

Interestingly, we identified several unique subsystem features of strain GG2 from RAST analysis (**Figure 1**). Similar to *Acinetobacter* ACICU and AYE strains, strain GG2 might utilize D-ribose and fructose as the major carbon source, besides having serine-glyoxylate cycle for one-carbon compound metabolism. This environmental bacterium also does not rely on siderophore in iron acquisition system, unlike other closely related *Acinetobacter* sp. Reflecting to its non-environmental lifestyle of strain GG2, the bacterium was found to encode less CDS for antibiotic-resistance compounds in comparison to pathogenic *A. baumannii* AB0057 and AYE strains. This is evident from the annotated genome that strain GG2 does not encode for aminoglycoside resistance genes as found in other strains. We also found several environmental important genes encoding extracellular lipase and cell wall-degrading enzymes such as endoglucanase. In metabolism of aromatic compounds, strain GG2 possesses genes for quinate degradation, a feature which was solely found in this organism. Similar to other *Acinetobacter* sp. except *A. baumannii* 19606, strain GG2 does not encode gentisate 1,2-dioxygenase responsible for degradation of xylenols and cresols. In contrast, genes involved in photosynthesis or motility and chemotaxis in strain GG2 and other *A. baumannii* strains were not identified in this study.

**FIGURE 2:**
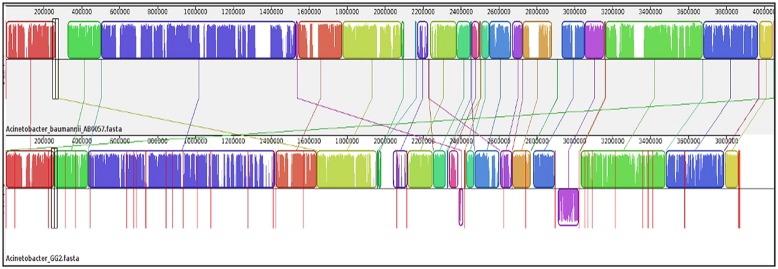
**Genome alignment performed using Mauve software between strain GG2 with its closest species, *A. baumannii* AB0057 (accession number: ABJM01000001) according to RAST analysis.** In this alignment process, a total of 38 locally collinear blocks (LCBs) with minimum weight of 133 were generated. Boxes with identical colors represent LCB, indicating homologous DNA regions shared between the two chromosomes without sequence rearrangement. Lines collate aligned segments between genomes. The vertical bars denote the conservation level, and upward and downward orientations relative to the genome line indicates collinear and inverted regions, respectively. Sequences outside colored blocks do not have homologues in the other genome. Red lines indicate contig boundaries within the assembly.

**FIGURE 3:**
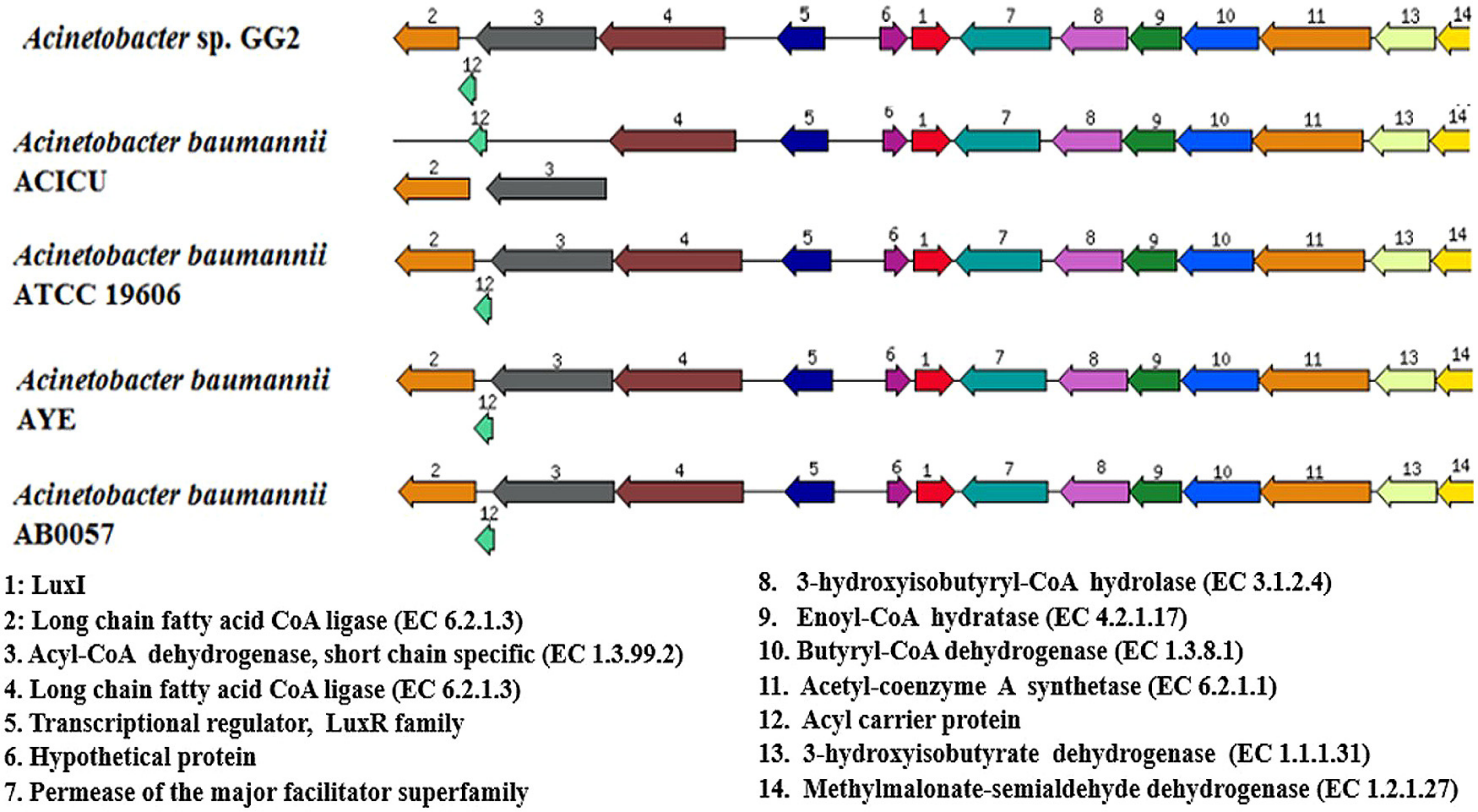
**The organization of LuxI/R homologues and their flanking genes in strain GG2 in comparison with closely related species, *A. baumannii* ACICU, ATCC 19606, AYE, and AB0057.** Genomes of respective strains are indicated on the left hand side. Homologous proteins are shown as the same color. Arrows indicate the relative orientations of the genes and genes which are located outside of the line indicates overlapping genes. All autoinducer synthesis proteins, together with transcriptional regulator LuxR homologue, were found on each strain.

Once gene annotation was performed, we were interested to look at the genome similarity between strain GG2 and other *Acinetobacter* species. Using BRIG software, the analysis of the genome sequence of strain GG2 showed high homology with at least 70% identity of the genome similarity with four closely related *A. baumannii* strains (Figure S1). Meanwhile, genomic alignments using MAUVE program indicated that strain GG2 shows a high degree of genome synteny with completely sequenced multi-drug resistant strain, *A. baumannii* AB0057 (**Figure 2**). The colored blocks of *A. baumannii* AB0057 are connected by lines to the homologous regions in the colored blocks of strain GG2. There are not many ‘white space’ which denote sequences not in homology blocks, hence showing the two species share a large number of their genome sequences. Areas that are completely white within a colored block are not aligned and they may contain sequence elements specific to a particular genome. The study on the genome assembly was then narrowed down to mainly CDS responsible for cell-to-cell communication among proteobacteria. Analysis of the LuxI gene clusters shows a conserved variation among strain GG2 with other close relatives of *Acinetobacter* sp. (**Figure 3**). All the *Acinetobacter* strains studied possess *luxI* homologues with presence of upstream transcriptional regulator, *luxR* homologues in reversed orientation. In the vicinity of the LuxI/R genes are long chain fatty acid coA ligase, acyl-CoA dehydrogenase, major facilitator superfamily transporter and enoyl CoA hydratase, all which are required in fatty acid synthesis.

**FIGURE 4:**
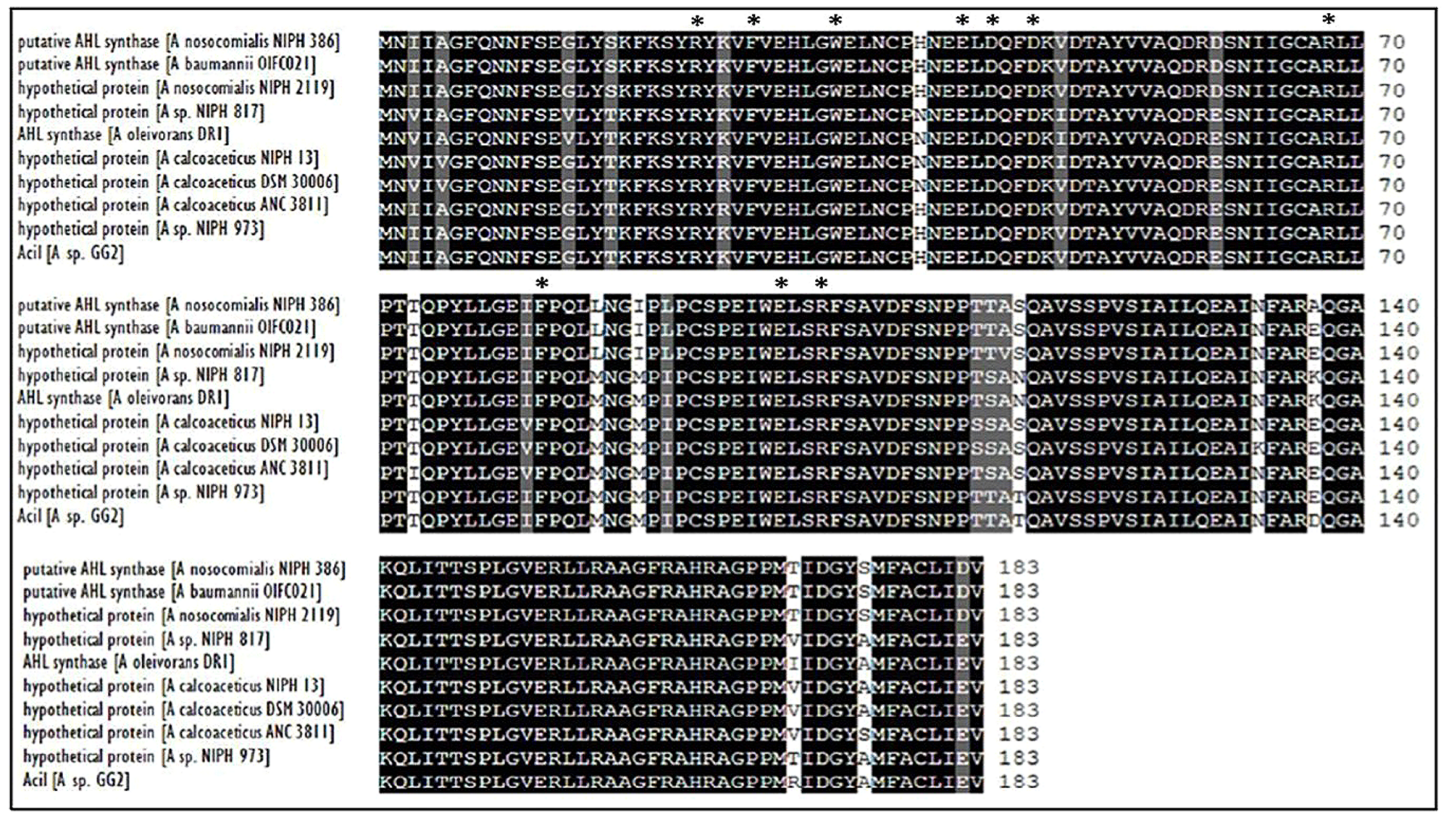
**CLUSTAL O (1.2.0) multiple sequence alignment of *N*-acyl homoserine lactone (AHL) autoinducer protein sequences of strain GG2 with protein sequences from other *Acinetobacter* species.** Sequences were derived from NCBI database (http://www.ncbi.nlm.nih.gov) and were aligned using CLUSTAL OMEGA software (http://www.ebi.ac.uk). Residues that are identical among the sequences are given a black background, and those that are similar among the sequences are given a gray background. The 10 invariant amino acids characteristics of LuxI homologues are denoted with asterisks. GenBank accession numbers (in parentheses): hypothetical protein from *A. nosocomialis* NIPH 2119 (WP_004711097.1), putative acyl-homoserine-lactone synthase from *A. baumannii* OIF021 (ELW82433), putative acyl-homoserine-lactone synthase from *A. nosocomialis* NIPH 386 (ENV42248.1), hypothetical protein from *Acinetobacter* sp. NIPH 973 (WP_004705984.1), hypothetical protein *Acinetobacter calcoaceticus* ANC 3811 (EOQ62174.1), hypothetical protein from *A. calcoaceticus* DSM 30006 (ENV97730.1), hypothetical protein from *A. calcoaceticus* NIPH 13 (ENU09934.1), hypothetical protein from *Acinetobacter* sp. NIPH817 (WP_004795350.1), AHL synthase autoinducer synthesis protein from *A. oleivorans* DR1 (YP_003734012.1).

**FIGURE 5:**
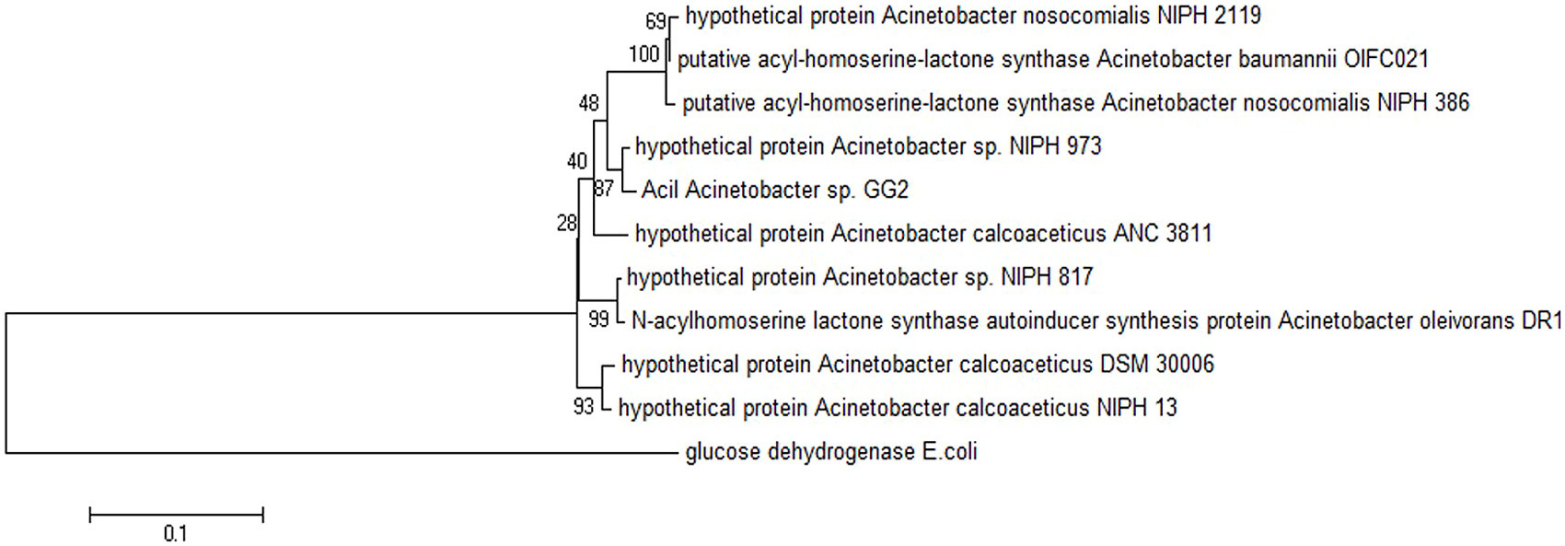
**Phylogenetic tree, generated using Neighbor-Joining algorithm, showing the phylogenetic position of the putative AHL synthase of strain GG2.** The tree is drawn to scale, with branch lengths in the same units as those of the evolutionary distances used to infer the phylogenetic tree. The horizontal bar at the bottom represents evolutionary distance as 0.1 change per nucleotide position. The numbers at the nodes indicate the bootstrap values as percentage of 1,000 replications. Glucose dehydrogenase enzyme (GI: 729809030) from *Escherichia coli* was chosen as the outgroup for comparison. GenBank accession numbers (in parentheses): hypothetical protein from *Acinetobacter nosocomialis* NIPH 2119 (WP_004711097.1), putative acyl-homoserine-lactone synthase from *A. baumannii* OIF021 (ELW82433), putative acyl-homoserine-lactone synthase from *Acinetobacter nosocomialis* NIPH 386 (ENV42248.1), hypothetical protein from *Acinetobacter* sp. NIPH 973 (WP_004705984.1), hypothetical protein *A. calcoaceticus* ANC 3811 (EOQ62174.1), hypothetical protein from *A. calcoaceticus* DSM 30006 (ENV97730.1), hypothetical protein from *A. calcoaceticus* NIPH 13 (ENU09934.1), hypothetical protein from *Acinetobacter* sp. NIPH817 (WP_004795350.1), AHL synthase autoinducer synthesis protein from *Acinetobacter oleivorans* DR1 (YP_003734012.1).

Web-based similarity searches against the GenBank database indicated that AciI protein sequence is highly homologous to other AHL synthase and several proteins from other *Acinetobacter* species with 96% sequence identity with AHL synthase from *A. calcoaceticus*. In addition, AciI protein shares 88% identical residues with several members of LuxI family from *Acinetobacter* sps. In fact, the multiple sequence alignments revealed that AciI protein shares high homology as well as the 10 conserved amino acids with other reported autoinducer proteins of *Acinetobacter* sps. as shown in **Figure 4**. On the other hand, the phylogenetic tree constructed based on amino acid alignment (**Figure 5**) illustrated that AciI was clustered closely with a hypothetical protein from *Acinetobacter* sp. NIPH 973 with bootstrap value of 87%. However, AciI was found to be the least phylogenetically related to AHL synthase from *A. oleivorans* DR1 and a hypothetical protein from *Acinetobacter* sp. NIPH817, possibly that these *Acinetobacter* species get diverted in evolution.

The putative AHL synthase, *aciI*, was found in contig 3 of the draft genome and its sequence has been deposited in the GenBank database (Accession number ALOW01000034.1). From NCBI database and Figure S2, this ORF encodes for a protein which consists of 183 amino acids. The sequence, TAAAG, at 32 nucleotides upstream from the start codon and the sequence, TTACCG, located at 60 nucleotides upstream correspond to the potential –10 and –35 transcription sequences, respectively. There are 17 nucleotides separating the two consensus regions, in agreement to the optimum spacing suggested by Hawley and McClure (1983) on *E. coli* promoter analysis. A putative Shine-Dalgarno site (AAGC) is located 8 bp upstream from the start codon while the transcription initiation site is postulated to be 7 bp downstream of –10 region. At the downstream part of the gene, there is a sequence likely to be a rho-independent transcription termination sequence from nucleotide 883 to nucleotide 1013 which consists of an inverted repeated sequence with the potential to form a hairpin structure with a 10-nucleotide loop and a 10-nucleotide stem including four pairs of CGs. Interestingly, a putative *lux*-box (CTGTAAATTTTTACAG) for strain GG2 was found 74 bp upstream of the start codon or immediately upstream of –35 element. This sequence is highly similar to the *lux*-box of *A. baumannii* M2 and was postulated to be the binding site for LuxR homologue protein, designated AbaR (Niu et al., 2008).

**FIGURE 6:**
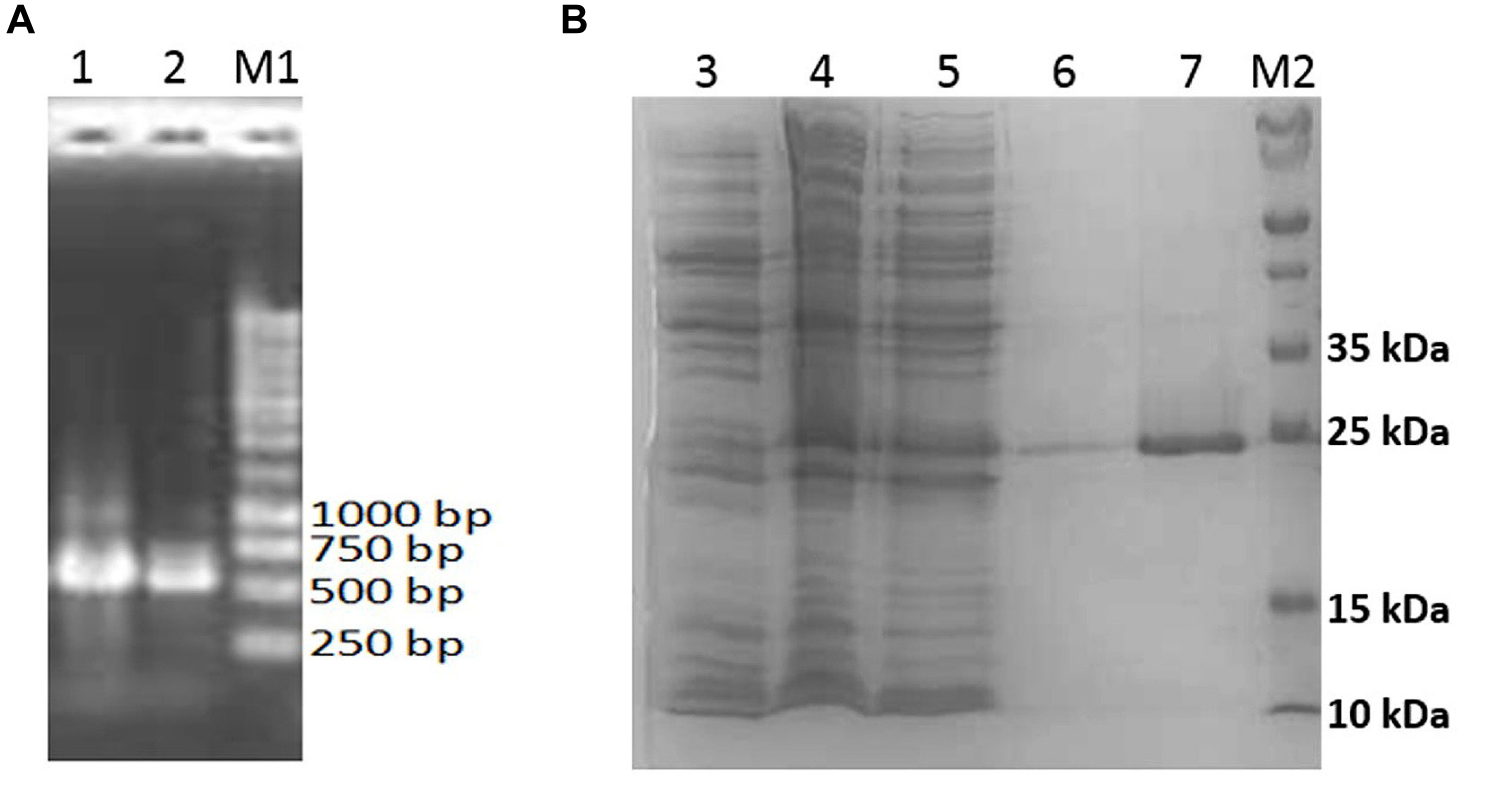
**Analysis of *aciI* gene and protein. (A)** Ethidium bromide-stained agarose gel containing *aciI* (gene amplification by PCR). Lanes 1 and 2 shows the amplified 552 bp amplicon. 5 μl of PCR products were loaded into each lane and electrophoresis was performed at 100 V. **(B)** SDS-PAGE analysis of the purified recombinant AciI protein. Lane 3, cell lysates of non-induced *E. coli* BL21 harboring pET28a-aciI; Lane 4, cell lysates of induced *E. coli* BL21 harboring pET28a-aciI; lane 5, flow-through fraction of purification step; lane 6, wash fraction of purification step; lane 7, eluted fraction containing recombinant AciI protein; lane M1, 1 kb DNA marker (Fermentas, Thermo Fisher Scientific, USA); lane M2, molecular weight markers (Bio-Rad, USA) with mass of each marker protein in kDa as indicated. The same amount of protein was loaded into each lane and subjected to electrophoresis at 150 V.

The 552 bp *aciI* was amplified by PCR (**Figure 6A**) and cloned into pET28a overexpression vector, producing pET28a-aciI, with a 6× His-tag driven by a T7 promoter. *E. coli* BL21 was transformed with this recombinant plasmid and the recombinant *aciI* gene was overexpresed upon IPTG induction. The His-tagged recombinant protein was later purified using affinity chromatography using nickel-chelated sepharose column (**Figure 6B**). By following the reading frame and prediction from ExPASy server (Wilkins et al., 1999), the theoretical isoelectric point (pI) of the recombinant protein is 5.37 and a molecular weight of 20.5 kDa. Nevertheless, the AciI protein expressed in *E. coli* cells was a fusion protein with 6× His-tag peptide, resulting in a protein with an estimated total molecular weight of 24 kDa. This is in accordance with the SDS PAGE profile of the purified recombinant protein as demonstrated in **Figure 6B**. Most of the recombinant protein was found in the cytoplasmic fraction of the cell lysate.

**FIGURE 7:**
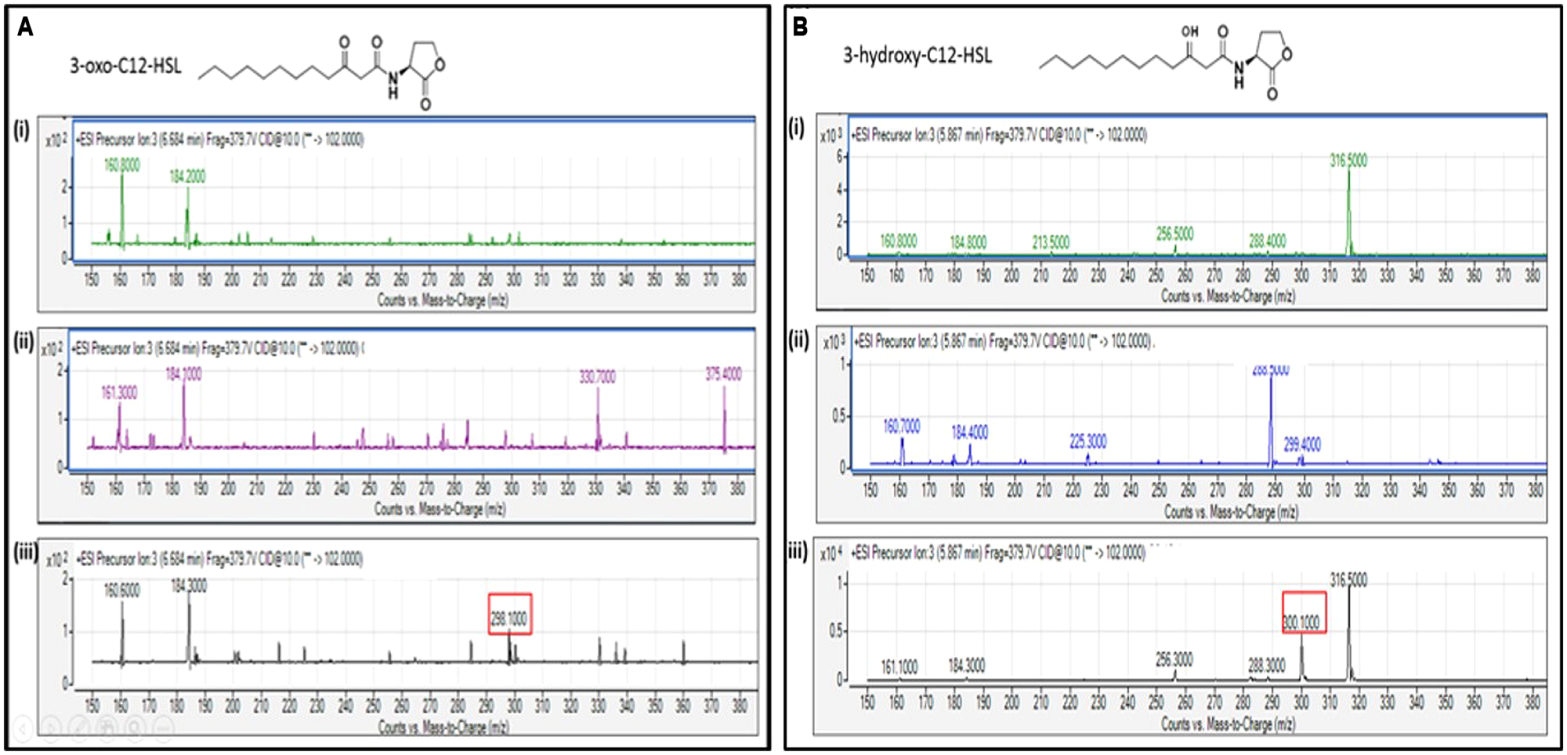
**Mass spectrometry (MS) analyses of the extract of spent culture supernatant from IPTG-induced *E. coli* BL21 harboring pET28a-aciI showing the presence of 3-oxo-C12-HSL and 3-hydroxy-C12-HSL.** By comparing with the corresponding synthetic AHL standards, the mass spectra demonstrated the presence of **(A)** 3-oxo-C12-HSL at *m/z* 298.1000 and **(B)** 3-hydroxy-C12-HSL at m/z 300.1000. The retention time for 3-oxo-C12-HSL and 3-hydroxy-C12-HSL are 6.684 min and 5.867 min, respectively. (i) Mass spectra of *E. coli* BL21 harboring pET28a alone (control); (ii) mass spectra of non-induced *E. coli* BL21 harboring pET28a-aciI (control); (iii) mass spectra of induced *E. coli* BL21 harboring pET28a-aciI.

The extracted AHL from the spent culture supernatant of the IPTG-induced *E. coli* BL21 harboring pET28a-aciI was analyzed using Agilent 6490 Triple-Quad LC-MS/MS system. High-resolution mass spectrometry analysis demonstrated the presence of long chain AHLs, 3-oxo-dodecanoyl-homoserine lactone (3-oxo-C12-HSL) and 3-hydroxy-dodecanoyl-homoserine lactone (3-hydroxy-C12-HSL) with *m/z* values of 298.1000 and 300.1000, respectively (**Figure 7**). The mass spectra of the extracted AHL were indistinguishable to the corresponding synthetic compounds at their respective retention times. Both AHLs were not found in the *E. coli* BL21 harboring pET28a alone or pET28a-aciI in non-induced state. The mass spectra also revealed quantitatively that 3-hydroxy-C12-HSL was produced more abundantly than 3-oxo-C12-HSL after 8 h of induction.

## Discussion

*Acinetobacter* spp. are ubiquitous in nature, and commonly present in the soil, water, sewage and sediment environments, indicating the profound adaptability of this genus in different niches. They are also known as effective degraders of alkanes and aromatic hydrocarbons (Throne-Holst et al., 2007; Fischer et al., 2008). In drinking water, these Gram-negative bacteria tend to form aggregates (Simoes et al., 2008). However, some *Acinetobacter* species including *A. baumannii* are pathogenic and multi-drug resistant strains. In fact, *A. baumannii* is historically regarded as an opportunistic pathogen in which its ability to cause diseases is determined by major deficiencies in the immunocompromised patients rather than the intrinsic virulence determinants of the infecting strains (Joly-Guillou, 2005).

In this study, we deciphered the draft genome of GG2 and comparative genomic analysis was performed with its closest sequenced relatives. According to RAST analysis, an important point worth noting is the high similarities between strain GG2 and *A. baumannii* AB0057, *A. baumannii* ACICU, *A. baumannii* AYE, and *A. baumannii* ATCC 19606. These four closest species are primarily associated with large outbreaks of nosocomial infections isolated from hospitalized patients (Manchanda et al., 2010). The 3.89 Mb genome of strain GG2 has G+C content of 38.4%, a value close to 40% which corresponds to that reported for other members of the *Acinetobacter* genus (Vallenet et al., 2008). The genome size of strain GG2 was slightly smaller than its closely related species as it encodes for less CDS responsible for basic survival needs of the soil isolate such as carbohydrate metabolism and some virulence factors. These virulence factors were found primarily in some multi-drug resistant *Acinetobacter* species but not in strain GG2. A study has been shown that human clinical isolate *A. baumannii* AYE was found to harbor 86-kb genomic island, a drug resistance island present in the majority of published *A. baumannii* genomes (Adams et al., 2010). Up to date, some of the non-pathogenic soil-living *Acinetobacter* sps. which have been completely sequenced are *A. oleivorans* DR1, *A. baylyi* ADP1, and *A. calcoaceticus* PHEA-2 (Jung et al., 2011). Similar to these environmental strains, GG2 was found to have a broad range of metabolic capacities as demonstrated by RAST analysis. With such feature, this rhizospheric bacterium is well-adapted in nutrient acquisition in soil and rhizosphere ecosystems. We also observed relatively high number of genes involved in expressing cofactors, vitamins, prosthetic groups and pigments, suggesting the ability of this bacterium to cope with various growth conditions and stresses in diverse ecological niches.

As for carbohydrate metabolism, besides ability to utilize fructose and D-ribose, it is postulated that strain GG2 is able to metabolize glucose using Entner–Doudoroff pathway (EDP). CDS encoding key enzymes for EDP such as gluconokinase (*gntK*) and phosphogluconate dehydratase (edd) were found from the draft genome. Other key enzymes such as 2-keto-3-deoxy-6-phosphogluconate aldolase (eda) and enzymes involve in the phosphorylation of gluconate (i.e., *gntP*) were not found, possibly the CDS fall in the sequence gaps of the genome. The presence of a number of CDS which encodes enzymes responsible for exopolysaccharides synthesis is another additional feature of a soil-living organism.

In addition, RAST analysis confirms the absence of high affinity iron-binding molecules called siderophore. Without such phenotype, it is postulated that strain GG2 depends on hemin transport system to scavenge iron from its environment. This may reflect a competitive advantage of the bacterium to obtain iron to thrive in different kinds of rhizosphere environment. A study by Yamamoto et al. (1994) reported that when *Acinetobacter* sp. invades the host, one of the mechanism of persistence and toxicity is the iron acquisition system, a likely contributing factor in its pathogenesis. This possibly explains the ability of strain GG2 to establish its niche in rhizosphere environment. On the other hand, a substantial number of genes were found to be associated with degradation of aromatic compounds, particularly on degradation of quinate, an aromatic plant compound. This is supported by the presence of quinate dehydrogenase which was found in the annotated genome. This physiological attribute enables the bacteria to degrade the metabolites synthesized by the host plants. Apart from this, analysis by RAST revealed the annotated genes were 48% of the subsystem coverage. Further annotation and bioinformatics analysis on hypothetical proteins could shed light on the functional roles of proteins with unknown functions and may reveal novel proteins that confer a fitness advantage to strain GG2 within the host rhizosphere. These proteins could be crucial in plant-microbe interaction as the role of strain GG2 as endophyte or phytopathogen in ginger rhizosphere remains unknown.

The genomes of strain GG2 was aligned with *A. baumannii* AB0057, *A. baumannii* ACICU, *A. baumannii* AYE, and *A. baumannii* ATCC 19606 using BRIG software (Figure S1). The high similarity among the *Acinetobacter* sps. indicates a close relationship among the bacterial strains. Hence, it is believed that the four *A. baumannii* nosocomial strains may possibly be evolved from environmental strains such as strain GG2 or they may share some ancestry relationship. Analysis by MAUVE (**Figure 2**) indeed showed a high degree of synteny between strains GG2 and its closest species, *A. baumannii* AB0057, in agreement with BRIG analysis. *In silico* analysis of the *luxI* gene cluster among strain GG2 and its closely related species showed conserved LuxI/R QS-related genes (**Figure 3**) among the environmental and nosocomial pathogenic strains. A point worth noting is the presence of fatty acid synthesis-related genes which are found at both upstream and downstream regions of LuxI/R homologues. Such profound feature was also reported by Kang and Park (2010) in LuxI/R gene cluster of *Acinetobacter* sp. strain DR1. This may indicate the use of metabolites of fatty acid biosynthetic machinery as the precursors for autoinducer proteins to form AHL lactone ring and the acyl group in strain GG2 and its close relatives (Val and Cronan, 1998). However, such mechanism remains unknown and requires further validation.

In this work, the gene for putative AHL synthase from *Acinetobacter* GG2, designated as *aciI*, has been successfully cloned and characterized in this study. The recombinant protein was fused with His-tag peptide to facilitate the purification of the protein. The purified protein was in agreement with the estimated size from SDS-PAGE analysis. The deduced protein sequence was highly similar and conserved to several AHL synthases from other *Acinetobacter* spp. Analysis of the draft genome sequences revealed that AciI is highly to be the only member of the LuxI family in *Acinetobacter* sp. GG2 genome as there is no additional gene that encodes LuxI homologue. The multiple sequence alignment and phylogenetic tree constructed (**Figures 4** and **5**) illustrated a high degree of homology and conserved regions among AHL synthases from other *Acinetobacter* spp. All the strains shared the 10 invariant amino acids which are characteristics of LuxI homologues (Parsek et al., 1997). This strongly indicates a low rate of random mutation for this autoinducer gene. It also shows that these proteobacteria share similar basic QS mechanism and gene regulation in AHL synthesis although they are responsible for different target genes.

A detailed analysis of both upstream and downstream sequences of *aciI* gene found that although both –10 and –35 promoter regions are not strongly conserved, the sequences meet the requirement of the typical *E. coli* RNA polymerase σ^70^ consensus promoter sequences (Hawley and McClure, 1983; Harley and Reynolds, 1987). In addition, the prokaryotic transcription termination sequences were present downstream of the stop codon. The presence of such features at the promoter and downstream regions of the ORF serve as strong indications that the *aciI* mRNA is likely a monocistronic transcript, and therefore is transcribed independently of other genes and artificial factors.

When *E. coli* harboring the recombinant *aciI* was induced with IPTG for 8 h and its spent supernatants was assayed with LC-MS/MS, the presence of both long chain AHLs, 3-oxo-C12-HSL and 3-hydroxy-C12-HSL was confirmed, suggesting the AciI is indeed the AHL synthase of *Acinetobacter* sp. GG2 (**Figure 7**). Such findings are in consistent with a recent study by Chan et al. (2011) which obtained the same AHL profile in *Acinetobacter* sp. GG2. The production of 3-hydroxy-C12-HSL was much higher than 3-oxo-C12-HSL, possibly indicating the important role of the former AHL in executing the physiological functions of the cells or expressing virulence factors.

In the past decade, the role of autoinducer proteins in *Acinetobacter* sp. was widely explored. One of the earliest studies on AHL synthase produced by *Acinetobacter* was conducted by Niu et al. (2008). The gene, designated as *abaI* (EU334497), was found in *A. baumannii* strain M2, a major human nosocomial infectious pathogen. The study demonstrated the importance of AbaI in normal biofilm formation for the bacteria to survive under unfavorable growth conditions. An *abaI* null mutant was shown to be impaired in biofilm forming capabilities by 40% after 16 h of growth in comparison to its wild type strain, and this was restored when AHL was supplied externally, indicating that there is a direct role of AHL molecules in biofilm development (Niu et al., 2008). Nevertheless, the mechanism of QS in contributing to the virulence and pathogenic potential in these bacteria is yet to be known.

The association of AHL and biofilm formation was first demonstrated by McLean et al. (1997). The study showed the production of bacterial AHL in aquatic biofilms growing on submerged stones, but was not present in rocks lacking a biofilm (McLean et al., 1997). In contrast to other QS systems, the AHL-mediated QS signaling system in numerous bacterial species appears to control genes associated with colonization of eukaryotes and this process was shown to be facilitated by bacterial biofilms (Costerton et al., 1999). A recent study by Anbazhagan et al. (2012) found that ∼60% of the clinical isolates of *Acinetobacter* spp. showed a significant biofilm formation with production of AHL molecules under prolonged period of incubation. In fact, Niu et al. (2008) reported a knockout mutant of *abaI* homologue was shown to have inhibit formation of biofilm. In another study, QS-regulated gene expression was shown to play a vital role in the metal tolerance of biofilms in *A. junii* strain BB1A. In the presence of natural or synthetic QS inhibitor, the growth of strain BB1A leading to the biofilm formation in metal-supplemented medium was significantly inhibited with a longer lag phase (Sarkar and Chakraborty, 2008).

Interestingly, many *Acinetobacter* spp. show some varying AHL profile. A study by Gonzalez et al. (2001) on *A. calcoaceticus* BD 413 (an environmental strain) and two clinical isolates from hospitalized patients demonstrated that multiple signaling molecules with autoinducer activity were detected in each *Acinetobacter* strain. In another study, a set of 43 *Acinetobacter* strains from nosocomial and environmental sources were studied and it was shown that 63% of the bacterial strains produced more than one AHL. However, none of the AHL signals could be specifically assigned to a particular species of the genus *Acinetobacter* (Gonzalez et al., 2009).

In a recent report, Bitrian et al. (2012) performed analysis of virulence markers on nine hospital and environmental strains of *Acinetobacter* sp. and found that all the strains studied secreted medium to long-chain AHLs. No short chain (C4–C6) AHLs were detected in any case. This is a distinctive feature of strains belonging to the *A. calcoaceticus – A. baumannii* complex. The pathogenic *A. baumannii* strain M2 has been shown to produce a major AHL molecule 3-hydroxy-C12-HSL, directed by autoinducer synthase *abaI*. Although five additional minor AHLs (e.g., unsubstituted C10-HSL, C12-HSL, 3-hydroxy-C10-HSL, unsaturated 3-oxo-C11-HSL, C14-HSL) were detected in culture supernatants of this strain, only one AHL synthase gene was identified, suggesting that this synthase has low specificity and is capable of synthesizing other QS signals (Niu et al., 2008). In another study, a LuxI homologue, termed as AqsI, was identified from diesel-degrading *Acinetobacter* sp. strain DR1 (Kang and Park, 2010). Similar to AciI, this AqsI protein consists of 183 amino acids and was found to secrete C12-related AHLs. This is consistent with the findings that C12-related AHL was a major QS signal in *A. baumannii* strain M2 (Niu et al., 2008). The study by Kang and Park (2010) also demonstrated that ability of *aqsI* mutant of strain DR1 in producing biofilm and degrading hexane were reduced remarkably. However, the restoration of the mutant phenotype was observed after the addition of free wild-type cell supernatant and exogenous C_12_-AHL, indicating the importance of QS in bacterial communication (Kang and Park, 2010).

The most studied *Acinetobacter* spp. are clinical isolates which are mostly isolated from hospitalized patients. Hence, the genetics and molecular biology available from the environmental strains of *Acinetobacter* spp. is not well-documented. Nevertheless, phylogenetic tree analysis (**Figure 5**) in fact showed that the environmental isolate (i.e. strain GG2) are clustered with many clinical isolates. The high similarities among the clinical and environmental strains highly suggest that the environment is an ideal habitat for opportunistic human pathogens, especially the nutrient-rich rhizosphere, the zone around roots that is influenced by the plants. According to Berg et al. (2005), the features that make a bacterial strain an efficient plant growth promoter (e.g., antagonistic properties, colonization ability) could also make it an etiological agent in bacterial opportunistic infections.

In this present study, the genome of strain GG2 is a significant addition of the genomic data from the *Acinetobacter* genus and offers different prospects into how closely related organisms are successful in different environments. As information of bacterial traits determining its ability in host plants colonization is still limited, detailed analysis of the genome sequence of strain GG2 will help to shed some light in prediction of the roles of the bacterium in the rhizosphere environment. In addition, the cloning and characterization of *aciI* as the homologue of AHL synthase of *Acinetobacter* sp. GG2 represents the initial step in elucidating the precise role and the molecular mechanism of the autoinducer system possessed by the bacterium. Among other things, this work now makes it possible to construct mutants with defective *aciI* to determine the roles of AHL in *Acinetobacter* sp. GG2, and to study the interaction of this LuxI homologue with molecules demonstrating anti-QS properties. As such, this work provides an impetus for further investigation of the relationship of AHL, QS and quorum quenching in this rhizospheric isolate. On a final note, data from further studies also helps to shed some light on the role of strain GG2 and its adaptation in its niche of rhizosphere environment.

## Author Contributions

KYH and KGC conceived and designed the experiments; KYH performed the experiments and analyzed the data; KYH and KWH wrote the paper; CLK, CKS, WFY and KGC edited and approved the manuscript.

## Conflict of Interest Statement

The authors declare that the research was conducted in the absence of any commercial or financial relationships that could be construed as a potential conflict of interest.

## Footnotes

1 http://www.ncbi.nlm.nih.gov/

2 http://www.ncbi.nlm.nih.gov/gorf/gorf.html

3 http://www.expasy.org/

4 http://www.ebi.ac.uk
