# Supplementary material for: Unravelling the genome of long chain N-acylhomoserine lactone-producing Acinetobacter sp. strain GG2 and identification of its quorum sensing synthase gene
Source: Front Microbiol. 2015 Apr 14;6:240. doi: 10.3389/fmicb.2015.00240 (PMC4396500; doi:10.3389/fmicb.2015.00240)
Supplement: Supplementary file 1 [file Table_1.PDF]

Table S1: Genomic coverage of several major subsystems found in *Acinetobacter* sp. GG2 and comparison to its closest relatives (*A. baumannii* AB0057, AYE, ACICU and ATCC 19606). The number of subsystem feature counts of each strain is shown in number and percentage (in parentheses).

Subsystem	*Acinetobacter* sp. GG2 genes (%)	*A. baumannii* AB0057 genes (%)	*A. baumannii* AYE genes (%)	*A. baumannii* ACICU genes (%)	*A. baumannii* ATCC 19606 genes (%)
Amino acids and derivatives	441 (16.8)	453 (16.3)	443 (16.2)	461 (16.7)	400 (15.0)
Carbohydrates	272 (10.4)	305 (11.0)	289 (10.6)	300 (10.9)	302 (11.3)
Cofactors, vitamins, prosthetic groups and pigments	226 (8.6)	207 (7.5)	200 (7.3)	224 (8.1)	255 (9.6)
Protein metabolism	209 (8.0)	247 (8.9)	246 (9.0)	243 (8.8)	245 (9.2)
RNA metabolism	162 (6.2)	173 (6.2)	173 (6.3)	170 (6.2)	151 (5.7)
Fatty acids, lipids and isoprenoids	154 (5.9)	171 (6.2)	179 (6.5)	188 (6.8)	162 (6.1)
Membrane Transport	131 (5.0)	116 (4.2)	108 (3.9)	117 (4.2)	119 (4.5)
Cell wall and capsule	122 (4.7)	111 (4.0)	108 (3.9)	115 (4.2)	103 (3.9)
