# Supplementary material for: Unravelling the genome of long chain N-acylhomoserine lactone-producing Acinetobacter sp. strain GG2 and identification of its quorum sensing synthase gene
Source: Front Microbiol. 2015 Apr 14;6:240. doi: 10.3389/fmicb.2015.00240 (PMC4396500; doi:10.3389/fmicb.2015.00240)
Supplement: Supplementary file 2 [file Image_1.PDF]

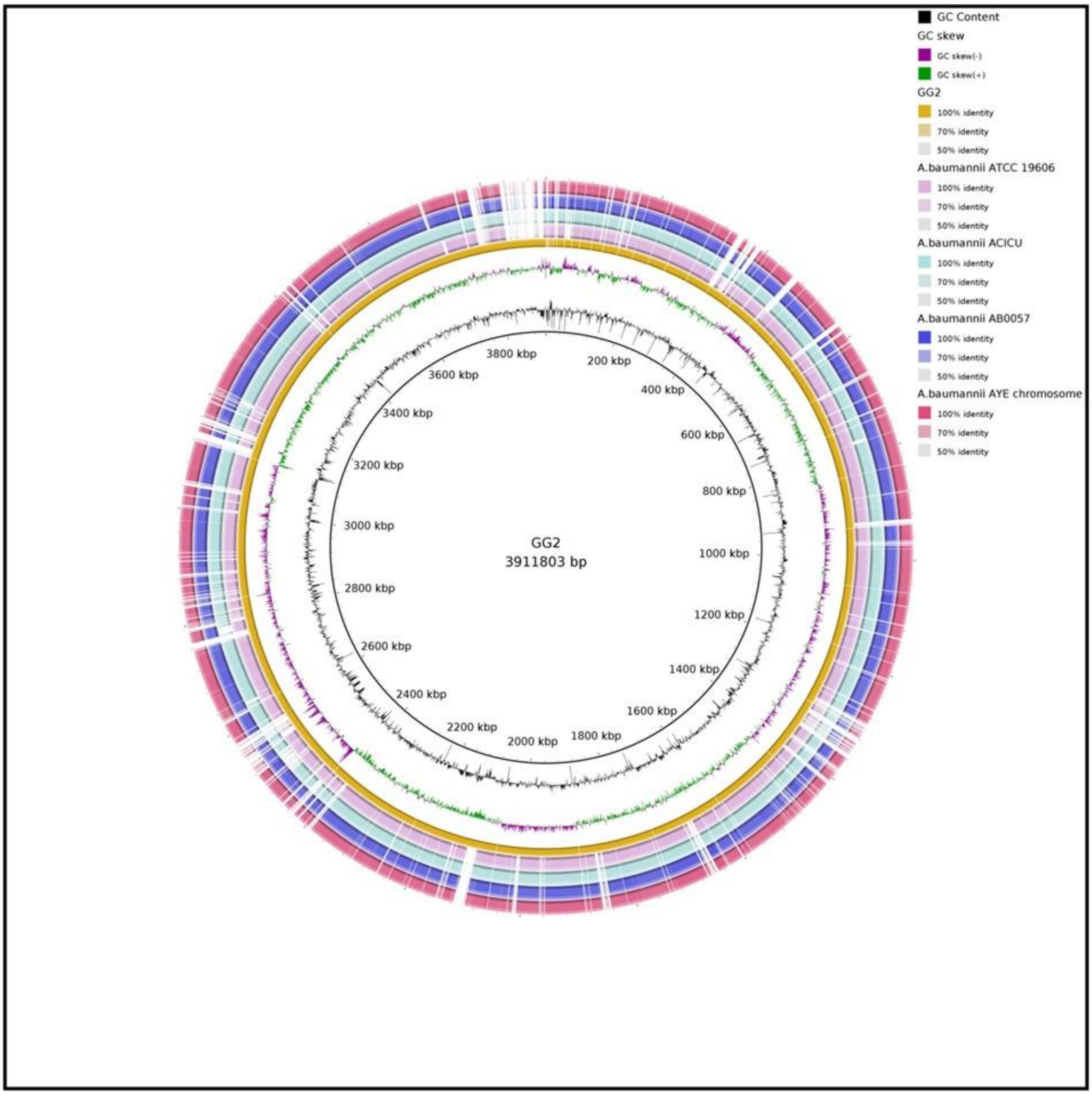

Figure S1. Circular maps from BRIG program comparing the genomes of strain GG2 (reference strain) with closely-related species, *A. baumannii* ATCC 19606, *A. baumannii* ACICU, *A. baumannii* AB0057 and *A. baumannii* AYE. The innermost rings represent GC content (Black) and GC Skew (purple/green) with respect to reference strain GG2 while the concentric rings of query sequences colored according to BLAST identity. The inner circle shows the scale (kbp).
