# Supplementary material for: Unravelling the genome of long chain N-acylhomoserine lactone-producing Acinetobacter sp. strain GG2 and identification of its quorum sensing synthase gene
Source: Front Microbiol. 2015 Apr 14;6:240. doi: 10.3389/fmicb.2015.00240 (PMC4396500; doi:10.3389/fmicb.2015.00240)
Supplement: Supplementary file 3 [file Image_2.PDF]

```
1    ACTGAATGGGCAACACTAAAGACCCCTGCTTTAAGTACTGGATGGGACTGGGTATTCATTGAGCAAAATGGGTCCGCTAGCTTAAAACGA   90
91   ATTGGCCTGCCGAGAAGCAATATCATGATCGTTGATGTATCTGGTACAGATATTGGATTTGATGTGACTGGATCATTAATCGAGAAGAAA  180
181  ATTGACAGCCTGTTCTGGGAACAGTTCATTTATGCCCAAATCAATACAAGATTAACAAAGTCTAAATTGAGCCCTAATTTCTCCTAGCAA  270
271  CCTGTAAATTTTTACAGTTACCGCCTTCTTAGCTGTTCAGTAAAGTCACATCAAAAATAAGTGGAAGCACTTGTAATGAATATTATTGCT  360
              lux box    -35                      -10                 S.D.        M  N  I  I  A

361  GGATTTCAAAACAATTTTTCAGAAGGCTTATATACCAAATTTAAAAGTTACCGCTACAAGGTGTTTGTTGAACATTTGGGATGGGAGTTA  450
      G  F  Q  N  N  F  S  E  G  L  Y  T  K  F  K  S  Y  R  Y  K  V  F  V  E  H  L  G  W  E  L
451  AACTGTCCACATAATGAAGAATTAGATCAATTTGATAAAGTCGATACAGCTTACGTCGTCGCTCAAGACAGAGAGTCCAATATTATTGGT  540
      N  C  P  H  N  E  E  L  D  Q  F  D  K  V  D  T  A  Y  V  V  A  Q  D  R  E  S  N  I  I  G
541  TGTGCCAGATTACTACCTACAACTCAACCGTATTTACTGGGTGAAATATTTCCTCAATTAATGAATGGAATGCCTATTCCCTGCTCACCA  630
      C  A  R  L  L  P  T  T  Q  P  Y  L  L  G  E  I  F  P  Q  L  M  N  G  M  P  I  P  C  S  P
631  GAAATTTGGGAACTCTCAAGGTTTTCTGCCGTAGATTTCTCGAATCCACCTACCACAGCGACTCAAGCAGTATCATCACCTGTTTCGATT  720
      E  I  W  E  L  S  R  F  S  A  V  D  F  S  N  P  P  T  T  A  T  Q  A  V  S  S  P  V  S  I
721  GCCATTCTGCAAGAAGCAATTAACTTTGCAAGAGACCAAGGTGCCAAACAACTTATTACCACCTCACCTCTTGGGGTAGAGCGGTTACTA  810
      A  I  L  Q  E  A  I  N  F  A  R  D  Q  G  A  K  Q  L  I  T  T  S  P  L  G  V  E  R  L  L
811  CGTGCTGCTGGGTTTCGTGCCCATCGTGCAGGCCCACCAATGAGGATTGATGGATATGCCATGTTTGCATGCTTGATTGAAGTGTAGTTT  900
      R  A  A  G  F  R  A  H  R  A  G  P  P  M  R  I  D  G  Y  A  M  F  A  C  L  I  E  V  *
901  GTTAAATCAAAGTCTAAGAATATTGGTGACATAGCAAAGATGAAGGTCTTAAGCCCGAATCTCGTTTCATTCGCTAAATTTCAAAAGCCC  990
991  TAGTCAAAAGATTAGGGCTTTTCTCTATTTGAAAGAATCAAACTTAATATTAAGACTGCTCAGTAATTTTGGTTTTCAAATTTAATTCTG  990
1081 ATTTCTTATGGTAAGCCTCATCAGTTTTAGTCTGCCAATCACGTGGCATAAATGAAGTCGATATAATCGCAACGATAGCCATAGATCCAA 1080
1171 TATAAATCATAATTAAGTAAGGATTACCCTGCCCGATACTTAAAAGTTTGGTCG                                     1224
```

Figure S2. The nucleotide sequence of *aciI* gene of strain GG2 and its flanking sequence. Single letters which code for deduced amino acid sequence are shown below the nucleotide sequence. The stop codon (TAG) is marked by an asterisk. The translational start site (M) is in bold and S.D. denotes the putative Shine-Dalgarno site. The proposed core promoter elements, -10 and -35 boxes, as well as the translational start site (A) are underlined and highlighted in yellow, respectively. The transcription terminator which was located downstream of the *aciI* is highlighted in green. A putative *lux* box was highlighted in grey.
